# Neural correlates of the natural observation of an emotionally loaded video

**DOI:** 10.1371/journal.pone.0198731

**Published:** 2018-06-08

**Authors:** Melanni Nanni, Joel Martínez-Soto, Leopoldo Gonzalez-Santos, Fernando A. Barrios

**Affiliations:** 1 Universidad Nacional Autónoma de México, Instituto de Neurobiología, Querétaro, México; 2 Department of Psychology, Universidad de Guanajuato, León, Guanajuato, México; University of Maryland at College Park, UNITED STATES

## Abstract

Studies based on a paradigm of free or natural viewing have revealed characteristics that allow us to know how the brain processes stimuli within a natural environment. This method has been little used to study brain function. With a connectivity approach, we examine the processing of emotions using an exploratory method to analyze functional magnetic resonance imaging (fMRI) data. This research describes our approach to modeling stress paradigms suitable for neuroimaging environments. We showed a short film (4.54 minutes) with high negative emotional valence and high arousal content to 24 healthy male subjects (36.42 years old; *SD* = 12.14) during fMRI. Independent component analysis (ICA) was used to identify networks based on spatial statistical independence. Through this analysis we identified the sensorimotor system and its influence on the dorsal attention and default-mode networks, which in turn have reciprocal activity and modulate networks described as emotional.

## Introduction

Understanding how the human brain processes information in a natural environment reveals information about its integral functioning during daily life. Under conditions simulating normal life certain areas of the brain function as networks participating in coding complex stimuli, such as natural vision [1]. This brain activity reflects characteristics similar to those evoked in the natural human environment. The vast majority of neuroimaging experiments to date sought to better understand the neural basis of perception using carefully controlled stimuli and tasks [1,2]. fMRI has been used to measure brain activity, primarily in the context of highly controlled experiments using purposefully design stimuli [3]. In these cases, researchers use pre-determined, static, and isolated-object images that are flashed on the screen in image acquisition synchronized paradigms [4]. Many contemporaneous experiments reduce the temporal complexity of their visual or auditory stimuli, presenting stimuli for one or two seconds or less. Yet, in order to sense and act in real-life circumstances, the brain must gather information over both long and short time intervals [5]. The use of precisely parameterized stimuli is critical for isolating the experimentally relevant dimensions from the extremely multidimensional natural stimuli.

Thus, in general, the world seen in the highly controlled fMRI experimental settings seems to bear little resemblance to our natural viewing experience [4,6]. Because many studies use simplified static stimuli, surprisingly little is known about how the human brain operates during real-world experiences. Exploring brain function with dynamic, naturalistic stimuli is important for several reasons. First, it is vital to determine whether results obtained in experiments using simplified stimuli hold true under natural conditions. Second, some research questions can only be addressed with naturalistic tasks where there is little temporal regularity [7]. For these reasons, using more natural stimuli helps to detect brain activation patterns that are difficult to observe using simple stimuli and enables us to study the human brain under ecologically valid, naturalistic stimulus and task conditions [1].

Naturalistic viewing paradigms (e.g. movies) have been used to study both stimulus-evoked BOLD signal changes and functional connectivity [8]. In order to preserve the dynamic sensory and affective qualities of real events, movies are used as tools closer to ecological vision in several studies [1,4,9,10].

The use of aversive cinematographic material has been reported as a means to induce brain related changes associated with acute stress [11,12]. Usually, the word stress is used to describe experiences that are challenging physiologically and emotionally [13]. Stress refers to a situation in which demands are perceived to exceed one’s personal resources. Different stressor types characterize different neurophysiological responses. For example, reactive stressors (such as pain) tend to implicate brainstem and specific hypothalamic nuclei, and the bed nucleus of the stria terminalis, which all have direct connections to the paraventricular nucleus [14]. On the other hand, anticipatory stressors (e.g. unfamiliar situations) seem to engage limbic system regions, namely the hippocampus, the amygdala, and medial prefrontal cortex [14]. Physical stressors would engage more heavily the limbic system (amygdala), while psychological acute stressors would emphasize the hippocampus [15]. Acute stress has an important impact on higher-order cognitive function, whose effects are believed to result from stress-induced alterations of large-scale brain networks [12]. Studies about the impact of stress on resting state networks (RSNs) show that stressed participants exhibited greater activation in the RSNs -Default Mode, Ventral Attention, Dorsal Attention, Primary Visual, and Sensorimotor networks- than non-stressed participants (Soares, Sampaio, Ferreira, et al., 2013a; Soares, Sampaio, Marques, et al., 2012b). Also, participants in a stressful condition have displayed deficiencies in the deactivation of RSNs vs. non-stressed participants [16]. Similarly, brain connectivity during acute stress is related to alterations in brain areas related to perception, vigilance, and deactivation of the Default Mode Network (DMN), suggesting the promotion of focused attention that optimizes threat detection [17]. Hermans et al. 2011, investigated changes in brain interconnectivity after exposure to a fear related acute stressor (through a movie clip), resulting in noradrenergic neuromodulatory activity that leads to a relocation of neural responses related to attentional reorienting, vigilant perceptual intake, and autonomic control findings that agree with a causal link between the salience network and stress-induced noradrenergic activity.

In Independent Component Analysis (ICA), the BOLD signal is treated as a mixed signal that is mathematically divided into several, statistically separate signals known as independent components. These independent components can then be related back to the stimuli to understand the relationship between brain activity and stimuli [7]. In fMRI studies, ICA has been used to extract networks of brain activity during resting state studies. In the field of emotions, functional connectivity has been used to estimate the stress on the connectivity networks [1]. There are few studies in which ICA has been used to estimate the neural networks in the course of emotion processing [18–21]. Also, ICA has been applied to analyze fMRI data collected during movie watching [9,10]. Specifically, there is a lack of studies that describe the connectivity networks associated with resting state conditions under a stressful or emotionally disturbing setting [22,23]. Through this perspective it is proposed that the connectivity patterns evaluated reflect brain-environment interactions rather than responses to stimuli [24] as observed in fMRI experiments on stress processing. Therefore, it is possible to consider the ICA approach as a valuable tool for revealing novel information about functional brain connectivity related with the stress processing phenomena.

Given the nature of the ICA approach, the present work proposes studying the neuronal bases of the emotional processes in natural and free conditions without establishing a controlled variable a priori. The aim is to validate an induced-stress method using functional neuroimaging paradigms. We propose an ICA connectivity approach to characterize the brain networks implicated during the view of a stress-inducing video in order to explore the brain networks implicated during the perception of an emotionally aversive condition. We also aim to identify the main connectivity networks and to elucidate their contrasts and the modulation that takes place during aversive emotional processing during a free-viewing paradigm. We approached this question by studying the functional organization of the human cortex when free-viewing a continuous sequence (4.54 minutes) taken from an original film. We reasoned that such rich and complex visual stimuli are much closer to an ecological vision than the controlled stimuli usually used in fMRI. Using behavioral tests, we also evaluated whether the video observation was a valid model for inducing psychological stress within an fMRI environment [25]. This video clip has been used in previous studies to induce acute psychological stress [26–28]. Likewise, the present stress induction method is consistent with the exogenous determinants of human stress response: unpredictability, uncontrollability and novelty [29]. Therefore, in the present study we also link the behavioral responses (perceived stress) derived from specific parts of said clip with increase connectivity in specific brain structures.

## Results

### Behavioral data

The results show the statistically significant differences in the groups before (t_1_) and after watching the stressful video (t_2_). The scores (*Mdn = 2*.*10*) at t_2_ are those with the highest stress subscale intensity (*Z* = -2.89, *p* = .*00*) when compared with t_1_ scores (*Mdn = 1*.*60*); this indicates that the stress-induction manipulation was successful. No statistically significant differences in perceived stress were found in the group control before (t_1_; *Mdn = 1*.*55*) and after seeing the movie´s segments (t_2_; *Mdn* = 1.66) *Z* = -0.56, *p* = .96 indicating no influence of the control stimulus in the stress levels.

### fMRI ICA data

An estimated total of 31 components were observed by ICA (31 spatial maps with their corresponding time course) and ordered by the extent to which they explain the total variation in data. Five components were excluded (C17, C23, C24, C28 and C29) because they contained elements characteristic of a typical artifact [4]. The explained variance percentage value for the first component was around 6%, and it decays for later components, with the value for the last component being 1.13%. On the other hand, in the average power spectrum of the time course of all the components, a predominant frequency was identified at 0.05 Hz, indicating the reliability of the components [30].

The common components resulting of the join activity show the variety of relative responses in the whole session/subject analysis. The components have been ordered according to the mean response per decreasing component, without considering artifact components (17, 23, 24, 28 and 29), and all adjustments were significant (*p* < 0.00) for all except 30 (*p* < 0.00278) and 31 (*p* < 0.00268); the significance test was not corrected. Therefore, the trustworthiness of each component is known with regard to its representation of the joint activity.

### Network dynamics

**Fig 1:**
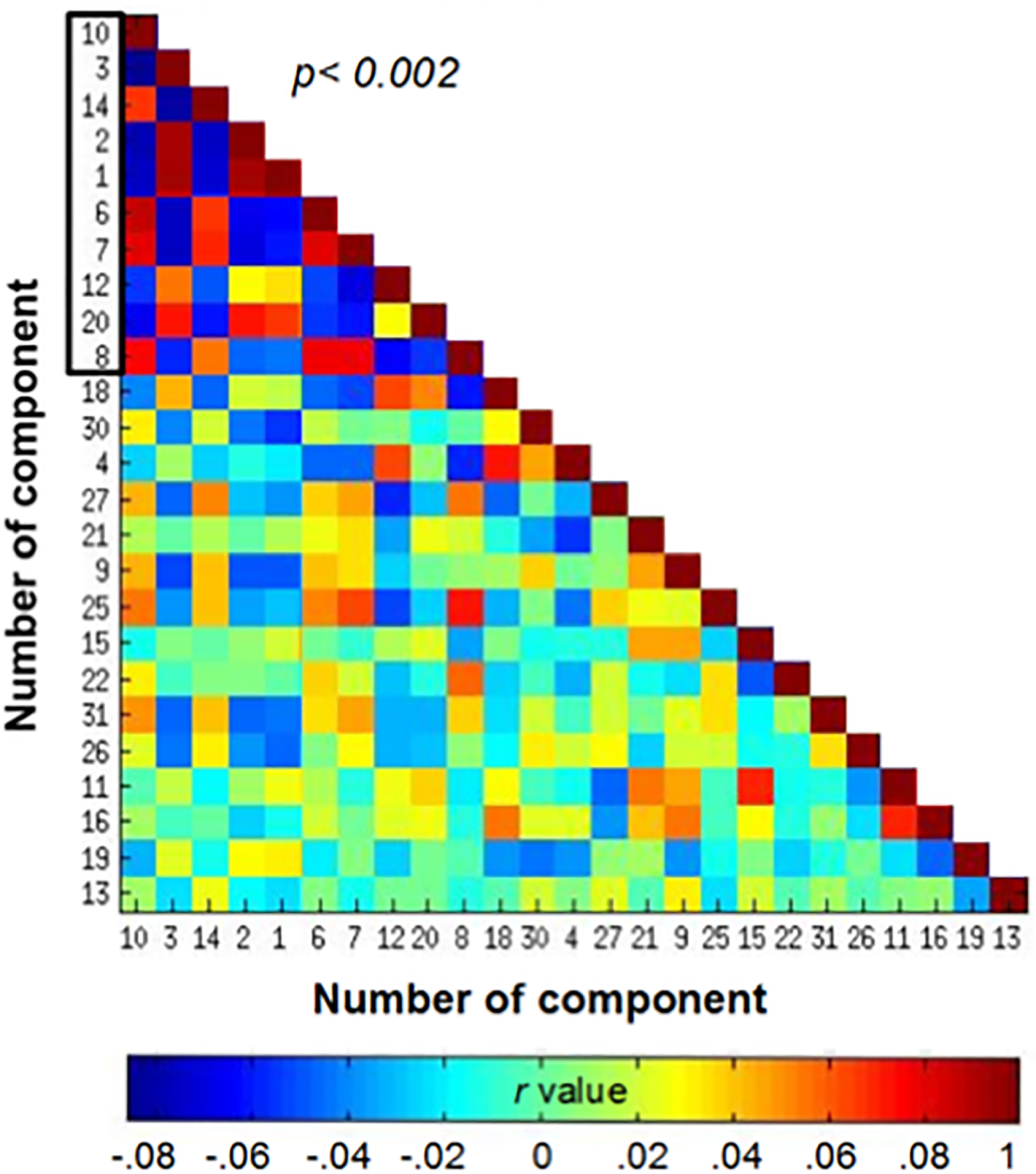
Correlation matrix of the BOLD signal. Correlation matrix of the BOLD signal time courses for the 26 ICA components. The matrix elements (I, j) represent the Pearson’s coefficient resulting from the cross correlation of the BOLD signal time course between the i and j components. The axes represent each of the component indices, the color scale shows the Pearson’s value. Associated network activity is depicted in Fig 2. Outline box contains the components which most of their correlation were significant (p<0.002 corrected) taken for further analysis.

**Fig 2:**
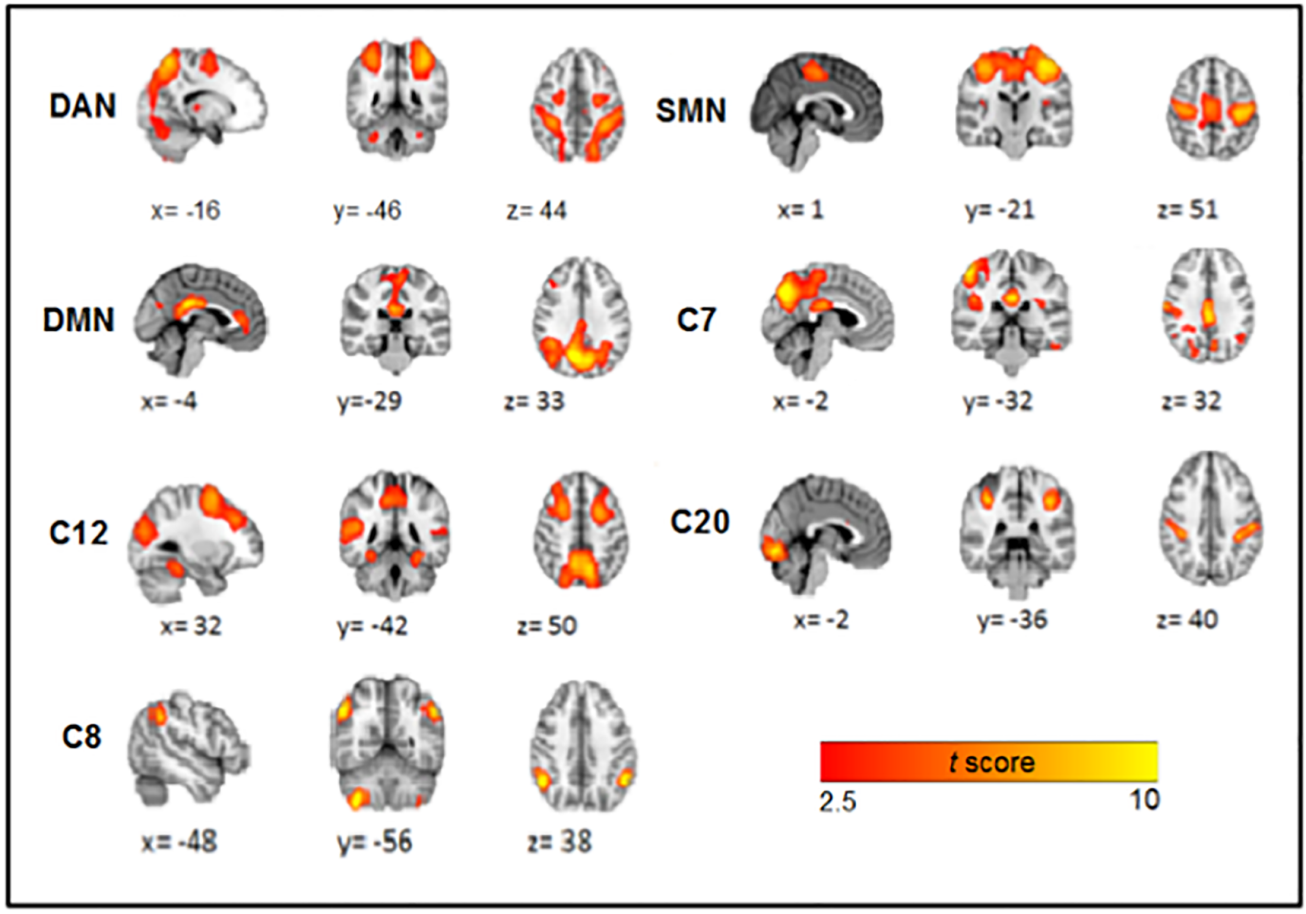
Brain networks included in the correlation matrix. Some of the networks taken from the correlation matrix in Fig 1 (outlined box) based on its order: A) C1, C2, C3 and C5; Dorsal Attention Network (DAN). B) C14; Sensory motor Network (SMN). C) C6; Default Mode Network (DMN). D) C7 posterior cingulate gyrus, lingual gyrus, precuneus. E) C12 Medial frontal gyrus, precuneus, medial temporal gyrus, fusiform gyrus. F) C20 Lingual gyrus, supramarginal gyrus, postcentral gyrus. G) C8 cerebellum anterior right lobe VIII-B area, angular bilateral gyrus.

**Fig 3:**
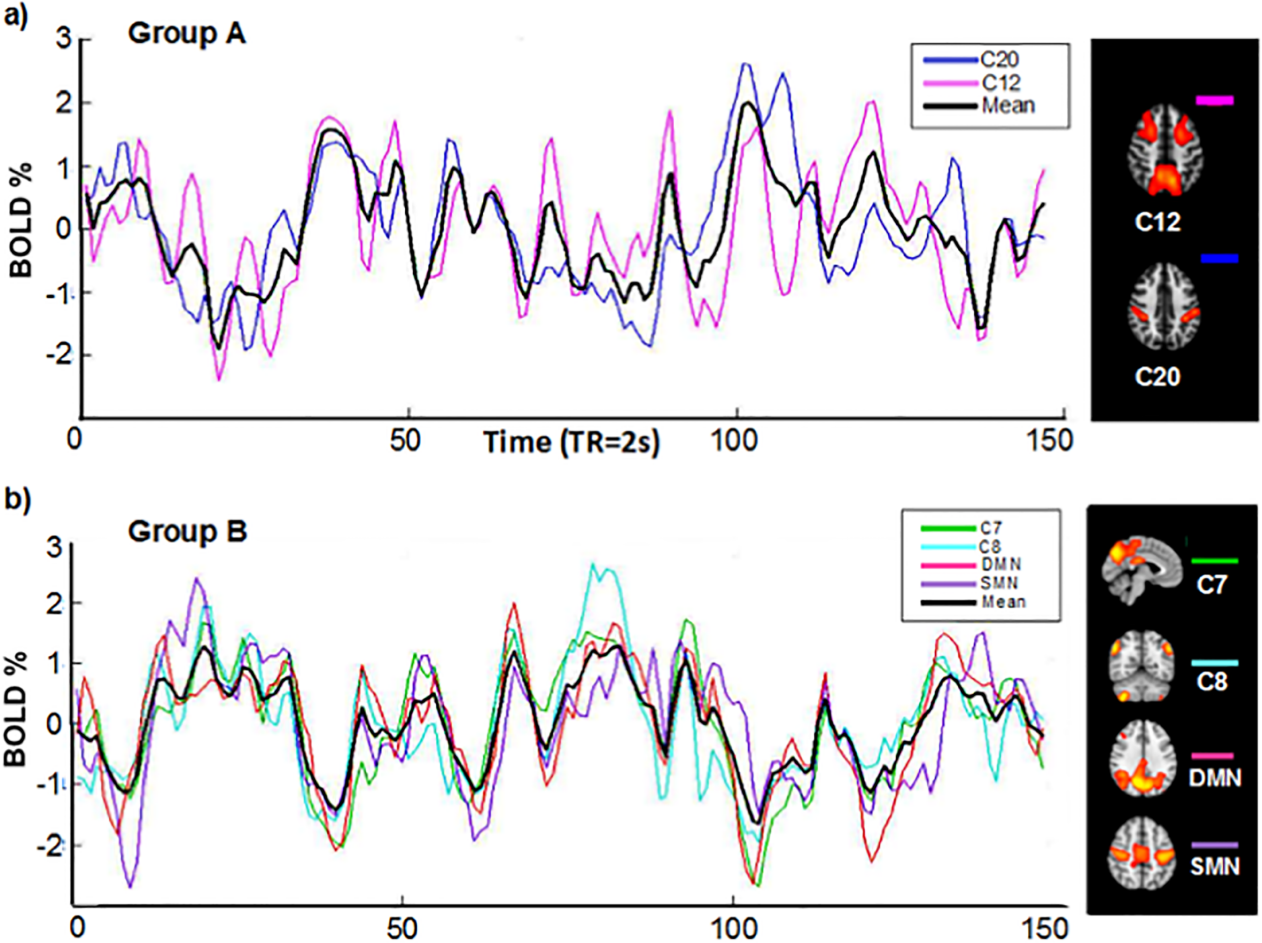
BOLD signal components with signitican correlations. Shown are components with significant correlation (see Fig 1). Average signals (black) obtained from BOLD signals. (group A mean = 0.1060 SD = 2.3151; group B mean = 0.1122 SD = 1.9606) a) Components 12 and 20. b) Components 7, 8, 10 and 14.

A matrix correlation was built from time courses of every component (27) (Fig 1). We took the components whose most correlations were significant among them *p*<0.002 (of a 0.05 Bonferroni corrected for 25 pairwise comparisons) for further analysis. These represent diverse networks. C1, C2, C3 and C5 were identified as the same network (dorsal attention network), by having a similar hemodynamic pattern in their spatial courses and having this high correlation. These four signals were averaged, obtaining a unique network for further analysis (Fig 2). Networks previously described as resting state [31], as sensorimotor (SMN), default mode (DMN) and dorsal attention network (DAN) (Figs 2 and 3) were identified. The networks represented by these components are shown in Fig 2.

**Fig 4:**
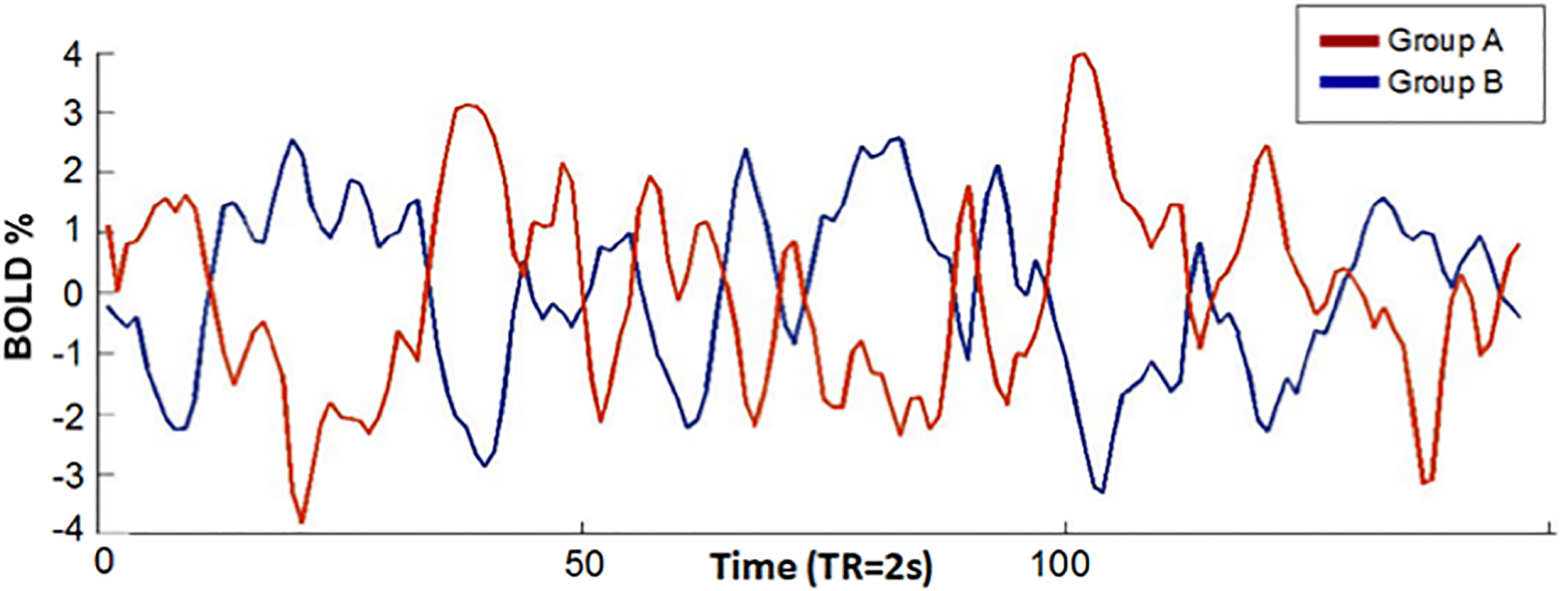
Plots of time signal components average. Two signal examples. The red line represents the average of component time courses 12 and 20 group A; the blue line of components 7, 8, 10 and 14, group B. The axes indicate the normalized BOLD signal (X) over time (Y).

We took the DMN and the DAN as time series references, since their activity has been better characterized in fMRI studies [32–36]. For demonstration purposes, two groups were formed based on their time courses. One group, comprising C12 and C20 (group A) has reciprocal activity to DMN. The other group, formed by C7, C8, C10 and C14, (group B), has activity reciprocal to DAN (Fig 3). The time courses of each group were averaged, resulting in two unique signals (group A mean = 0.1060 SD = 2.3151; group B mean = 0.1122 SD = 1.9606). By splicing these signals, a remarkable negative correlation between them was observed (*r* = -0.8452, *p*< 0.01) (Fig 4).

**Fig 5:**
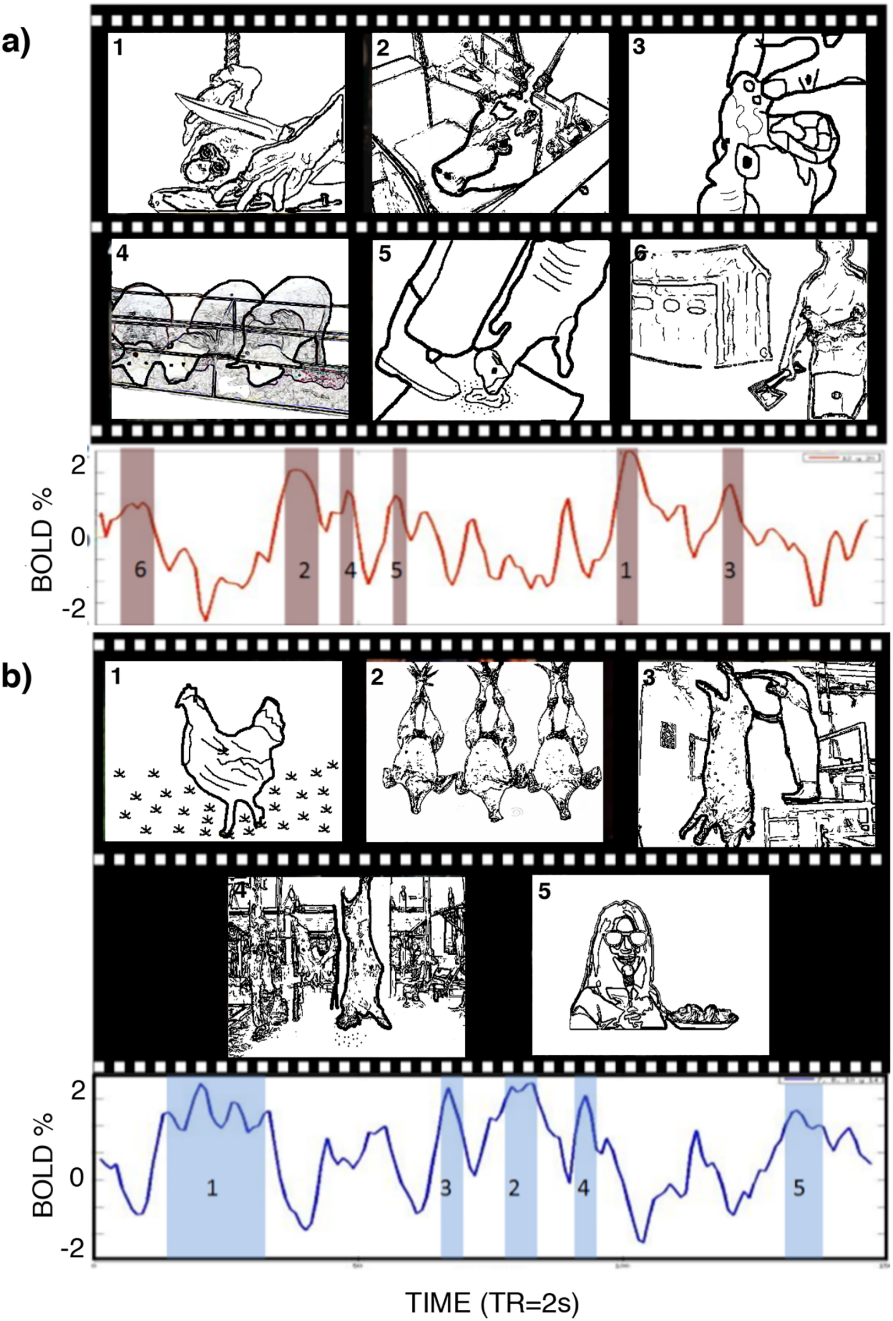
Types of scenes corresponding to high BOLD activity. A) Scenes corresponding to the highest BOLD signal peaks, from group A (C12 and C20 signal); these images are related to the slaughtering actions and are images where the animals are prepared to be hurt. B) Images from group B (C7, C8, C10 and C14 signal) while the animal is hurting (in the dying process). Note that due to copyright reasons, the images showed in this figure are not the actual images used in the study. However, all the images used in this figure represent similar situations observed in the original video. All the images used in this figure were constructed from images acquired and processed in our laboratory.

As we know, changes in these time courses depend on the video display; thus, we asked what movie frames elicited the highest activation in these two signals. Fig 5 shows the movie frames from the highest activation peaks. The frames were ordered according to descending signal amplitude. Group a) was triggered mainly by scenes from the time prior to hurting the animal, whereas group b) was activated by scenes where the animal was dying.

**Fig 6:**
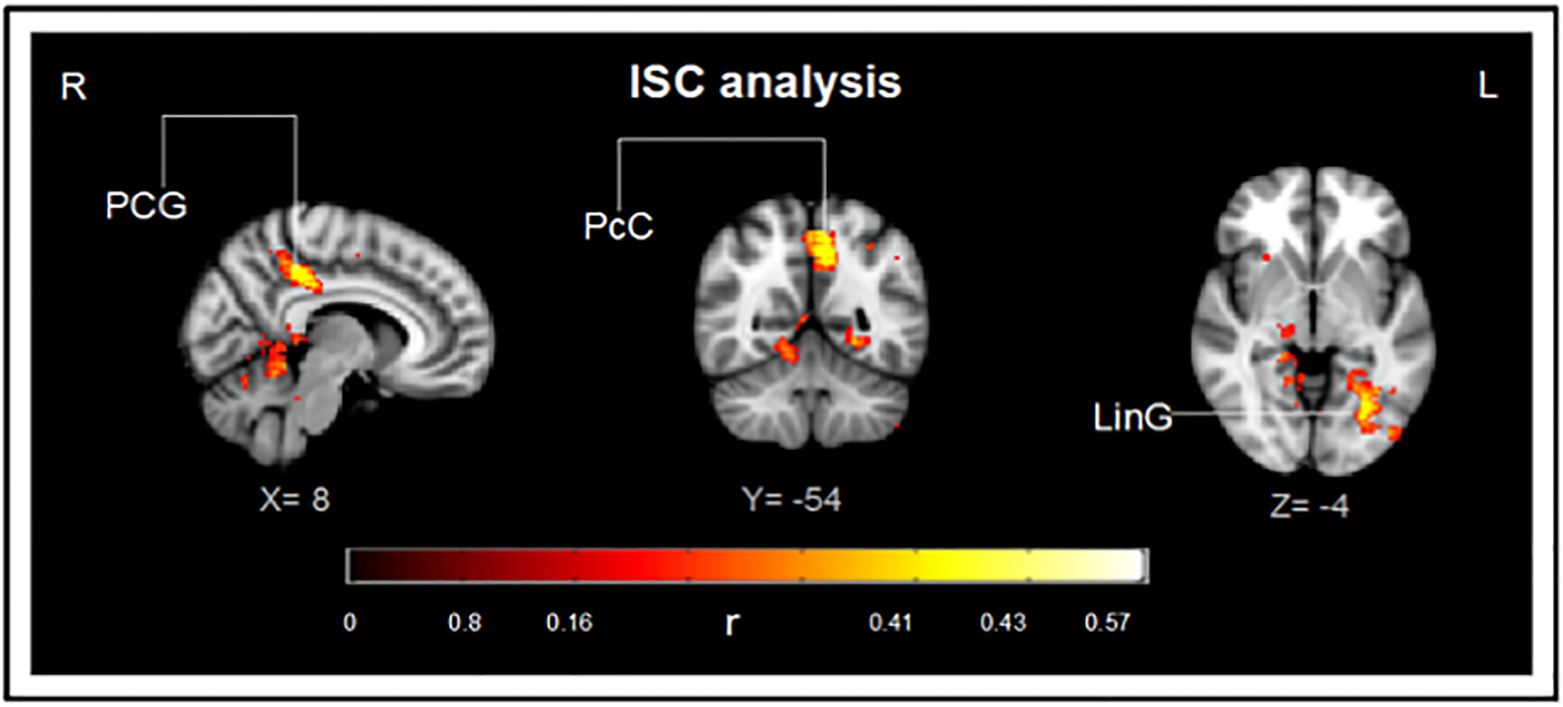
Areas with higher correlation. Areas with higher correlation between subjects during the visualization of the video. Precuneus surface cortex, fusiform gyrus, lateral left occipital areas, lingual gyrus, and posterior cingulate cortex.

Intersubject correlation analysis (ISC) revealed that the high correlation is mostly present in posterior areas, mainly the precuneus surface cortex, fusiform gyrus, followed by the lateral occipital areas, lingual gyrus, as well as parts of the dorsal limbic system (Fig 6).

We can observe two clusters with higher correlation, the precuneus cortex and the lingual gyrus. That means the activity in those areas was present in a most synchronized manner in all subjects across the time. Comparing with ICA results the lingual gyrus appears in three of the networks (components 7, 10 and 20). Precuneus is part of component 7, also an important part of the DMN, together with the posterior cingulate cortex, involved in visual, sensorimotor, and attentional information.

### Cross correlation

**Fig 7:**
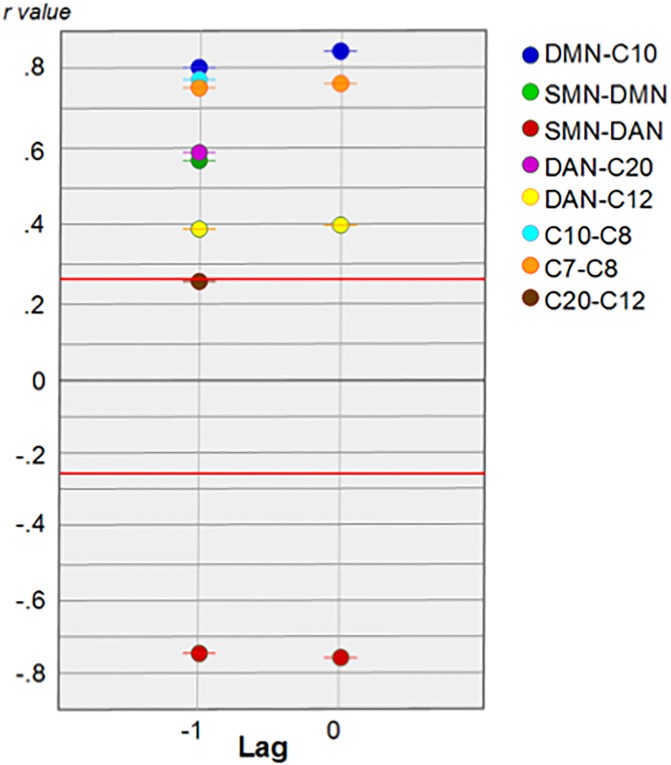
Highest values of cross correlation analyses. Results from cross correlation analysis showing the highest values for each comparison. For some (DMN-C10, SMN-DAN, DAN-C12, C7-C8) it shows also the next to the highest values. Eight comparisons, from which seven were significant (p<0.0125 corrected), the red lines correspond to the upper and lower confidence bounds.

**Fig 8:**
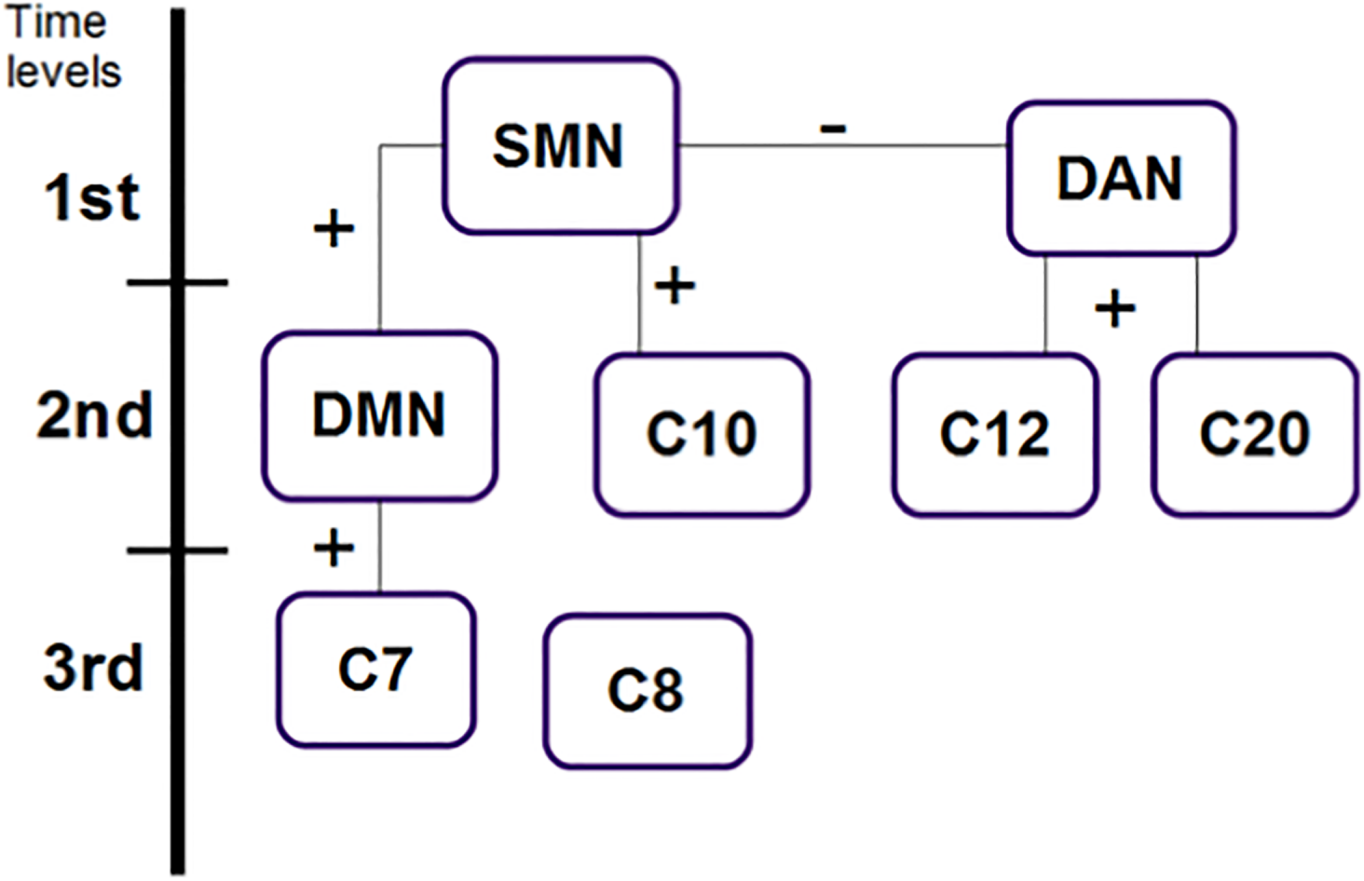
Network organization in temporal delay and correlation. Networks organized based on the temporary delay and correlation (positive or negative) that exist amongst them, according to Fig 7. They are set out in levels according to their activation point in time.

The results of cross correlation analysis were significant in most of comparisons p<0.0125 (of a 0.05 Bonferroni corrected for 8 pairwise comparisons) with estimation error bounds 2.7 standard deviations from 0, (Fig 7), show a tendency for most of the networks to be one lag against the other. As it shows DMN vs C10, SMN vs DAN, DAN vs C12 and C7 vs C8, their highest value is in lag 0, however the next value is in lag -1 which indicates a tendency to be ahead, i.e. DMN tends to be ahead C10 but not in a fully way. Standing out among these findings is the SMN, which predict directly the group of networks on the left side (DMN), and in an inverse way those in the right column (DAN) (Fig 8). These two networks positively predict other networks, whose areas include posterior cingulate gyrus, lingual gyrus and precuneus; b) anterior cingulate cortex, lingual gyrus, postcentral gyrus and posterior insula; and c) SMN, whose connectivity changed immediately after the animal-slaughtering scenes were shown. On the other hand, areas of networks with increased activity during scenes that predated the time of death were a) medial frontal gyrus, precuneus, medial temporal gyrus and fusiform gyrus; and b) lingual gyrus, supramarginal gyrus and postcentral gyrus.

## Discussion

The present study was designed to locate the main networks, their dynamics, and the modulation that takes place in aversive emotional processing using model free neuroimaging analysis [11]. Using ICA we were able to document the dynamics that exist between the BOLD activity of each network detected while a stressful video was being viewed. What stood out the most in this study was the identification of the DAN and the DMN. Our findings are consistent with previous research, especially the determination that these networks are intrinsically inversely correlated [37,38]. Furthermore, the signal of each network is also associated with the reciprocal activity of other networks among the six networks whose areas have been associated to emotional processing. Based on the correlation of their time courses, these six networks formed two subgroups, where the average signal of each subgroup had a high negative correlation value (r = -0.8452). The first subgroup, made up of components 12 and 20, contained networks that involve regions, such as the medial frontal gyrus, precuneus, middle temporal gyrus, fusiform gyrus, lingual gyrus, supramarginal gyrus, and postcentral gyrus, that have reciprocal activity with the DAN and are observed in moments of preparation before killing an animal. This response coincides with processes associated with this specific network, which include expectation and fixed attention when knowing that something is going to happen soon, marking a striking difference from other processes related to attention [39]. This activity begins to decline the moment the expected event occurs. From this point on, activity begins for the following group of networks. The ICA components 7, 8, 10, and 14 include regions 7 (posterior cingulate, lingual gyrus, and precuneus), 8 (right frontal lobe of the cerebellum area III-B and bilateral angular gyrus), 10 (anterior cingulate, lingual gyrus, post-central gyrus and posterior insula), and 14 (sensorimotor system, pre- and post-central gyrus). The corresponding BOLD activity involves the DMN. Despite the fact that the DMN is characterized as being present mostly during a resting state, its activity also persists during states of sensory processing that impose a minimum cognitive demand [40,41]. A modular focus of the brain tells us about its capacity to assemble quickly and robustly, in order to segregate an infinity of processes. This idea has been developed more tangibly using computer mechanisms that demonstrate its high efficiency and functioning by modulating multiple processes of complex information and developing tasks in a changing environment [42]. The main networks for the modular control on a resting state brain, have been demonstrated before and include the occipital, sensorimotor, and default mode networks [43,44]. These same networks were found at the hierarchical level of modulation [45], whereas at the voxel level researchers found visual, auditory, and default mode networks. As was made visible in our results of crossed correlation, the SMN precedes both of these groups and its activation in the absence of pain or physical sensation is associated with the perception of others’ pain, as suggested by other studies [46]. At the moment when the before-injury scene starts in the movie, DAN is fully activated, at the injury scene moment SMN begins to activate and DAN begins to deactivate. Afterwards, this same network (SMN) gives way to the DMN (after injury scene), which modulates other networks with areas such as the lingual gyrus, which has numerous connections with the amygdala, central lobe, (associated with the perception of unpleasant stimuli), the anterior and posterior cingulated cortex, part of the limbic system, cerebellum, postcentral gyrus, and angular gyrus.

The basic brain processes are the result of a series of events that occur in certain pathways through in the brain. For a stimuli processing in a complex environment, which combine top-down / bottom-up process, is necessary a network which emphasizes the behavior of a complex system probably shaped by the interactions between its constituents [47]. We think this is the first description of a path that operates in a complex network environment in response of complex stimuli. The present study also showed that the same modulating networks are maintained in both a stable state [44] and a stimulus-triggered altered state, leading to the possibility that these networks act as part of the general modulation of brain activity, regardless of the stimulus or process. However, this conclusion must be confirmed by studies made under the same model with different types of stimuli that provoke responses related to other cognitive processes. There are some studies with positive valence movies used in naturalistic behavioral paradigms [48]. However, the studies of processing of stress during free movie viewing are scarce [11]; movie film with interpersonal violence evaluated with neuroendocrine and affective measures of affect; with Salience Network related activation).

Here we have chosen a negative valence movie in order to create an ecological valid model for studying some neurocognitive process elicited by an acutely stressful experience [29]. Some alternative explanation for the amygdala activation absence could be considered, including: a) the variability of type of stressors and its engaging physiological and psychological activity [14]. Psychological acute stressors show high variability on the physiological systems [15,49]. b) psychological stress commonly involves ambivalent signals that require considerable integration and evaluation before a response stress has been started [15]. Since there is not much previous evidence of emotional studies with a free viewing stimulation, some of the results presented here lead to interpretations and questions of brain activity. One of them is the absence of amygdala activity in spatial maps, since its activity has not been reported in the analysis of long time windows under emotional stimuli, it can be considered the fact that there is no significant sustained temporal activity of it over other areas / networks, when viewed in a longer time window than those commonly used in fMRI block studies. This evidence has been proven only with dynamic stimuli [50,51]. We have explored the human brain’s response under stress induced by a dynamic naturalistic stimulus. Our technical and methodological approach differs from previous studies of psychological and physical stress paradigms suitable for neuroimaging environments [52,53]. Further, using more natural stimuli will probably allow us to detect patterns of brain activation that are difficult to observe using simple stimuli, and enable us to study a “stressed human brain” under ecologically valid naturalistic stimulus and task conditions [1]. Future studies would consider additional measures (physiological) of stress together with this method.

## Methods

### Participants

Twenty-four right-handed healthy male subjects were enrolled in the study. All subjects gave their written informed consent, and the experimental protocol was approved by the Bioethics Review Board of the Institute of Neurobiology, UNAM, and was performed in accordance with the Declaration of Helsinki. Subjects had an average age of 36.42 (*SD* 12.14) and a minimum of 12 years of schooling. All subjects were mid-to-high socioeconomic level. Individuals who met any of the following criteria were excluded: history of head injury, treatment with psychotropic medications, use of narcotics, steroids, or any other medication that affects the central nervous or endocrine systems, having had a medical illness within 3 weeks prior to testing, self-reported substance use or mental disorders (SCL R-90 [54]), daily tobacco use, regular nightshift work, current stressful episode or major life event, and regularly viewing extremely violent movies or playing violent computer games. One subject was excluded during the analysis due to numerous motion artifacts. Women were excluded from this study since the hormonal cycle may influence emotional responses and may cause a bias in the study [55].

### Stimuli and procedure

Prior to entering the scanner, subjects received thorough instructions about the scanning procedure and the tasks to perform. Perceived stress (aversive stimuli) was tested immediately after entering the scanner to obtain a baseline level (t_1_) and again after viewing 4.54 minutes of the stressful movie (t_2_). To show that the results are specific to our particular experimental condition and do not merely reflect generic modulations in the audio-visual stimulation, a control experiment was carried out with a comparable set of "neutral" movie segments in an independent group of subjects.

### Psychological measures

#### Perceived stress

The computerized version of the Stress and Activation Adjectives Checklist-SAACH [56] was employed, considering only the stress dimension (11 adjectives with four points answer format: absolutely true, probably true, not sure and absolutely not). In the present study the scale was adapted to be administered in the scanner environment. To respond to the test, subjects had a magnetic resonance (MR) compatible ResponseGrip (NordicNeuroLab) with their left and right index fingers and thumbs each placed on one of four buttons. All sentences and instructions were presented on a graphical interface programed in house where participants were instructed to rate their present emotional state, all the items presented were randomized. The sentences were printed in lower case letters, displayed in approximately 18-mm tall Arial, white on a black background, providing high legibility. Prior to the scanning session, each subject was instructed in the performance of SAACH and the use of the computer mouse by practicing a shortened version outside of the scanning environment on a desktop computer with verbal instruction from the researcher.

### Aversive stimuli

We use fMRI coupled with the eye tracking technology to confirm attentive viewing of all projected stimulus and movie fragments as suggested by other studies [57–59], where it is recognized that the monitoring of eye movements during fMRI is something essential in research on natural stimulation paradigms [60]. The eye tracker was calibrated for each participant before the experiments began. A MR-compatible NNL eye-tracking camera was used to record video data of the subject’s eyes during the fMRI task-related scanning (Visual System NNL EyeTracking Camera, and ViewPoint eye tracker software, Arrington Research Inc., Scottsdale AZ). Subjects watched 4 min and 54 sec. of the movie “Faces of Death #1” (dir. John Alan Schwartz, 1978, original length 105 min) in the fMRI scanner. Three short movie fragments were used to create the proper context (1 × 60s, 1 × 120s, 1 × 114s). This shorter version was re-edited with computer software. Selected fragments were comparable in amount of speech, human presence, luminance, and language. The aversive movie clips contained scenes of a woman on a farm decapitating a rooster, images of a slaughterhouse where sheep and cattle are being sacrificed in a brutal manner and video fragments of a group of actors pretending to eat the brain of a monkey that they had to kill themselves. Participants were instructed to carefully watch the video and feel free to stop the video if the images were disturbing them. Participants were informed before the experiment that watching the film might be stressful and that they could terminate the experiment at any point. The video clip image contents were classified with negative valence and high arousal content according to Bradley and Lang [61].

### Functional magnetic resonance imaging

During projection of the video, the sequence of images was captured with a 3.0 Tesla Discovery MR750 MRI unit, using the 32-channel reel for cranium, at the Neurobiology Institute of the UNAM. The functional images were acquired with a sequence of Eco Planar (EPI) pulses for heavy images at T2*, GE-EPI of TR / TE = 2000 / 40 ms in a 64 × 64 matrix over a FOV of 25.6 cm in 36 slices with a width of 4 mm per slice. This resulted in isometric voxels with a spatial resolution of 4 × 4 × 4 mm^3^. The high-resolution structural images were acquired using a T1 weighted SPGR pulse sequence with 1 × 1 × 1 mm^3^ of spatial resolution.

### Data analysis

#### fMRI data

All data were transferred to offline work stations to convert them from DICOM to NIfTI format (dcim2nii, Ch. Rorden, http://www.nitrc.org/projects/dcm2nii); afterwards they were processed using the MELODIC ICA module of the FSL program, which runs an algorithm of independent components per voxel in the stack of images for the whole group of subjects. All the functional studies were processed using the typical pre-statistics pipeline that includes timing correction for interleaved slice acquisition, for motion correction, normalization to standard space MNI with a 12 degrees linear transformation, resampled to 2mm spatial resolution, finally a smoothing of 6mm Gauss kernel at FWHM. The statistical analysis was estimated in to steps, single- and multi-session. The first analysis was made using the Single-session ICA mode that makes up a standard of each entrance file (23 subjects) to a two-dimensional matrix (time and space) in order to decompose them later into two-dimensional matrixes (time and components, space and components). The result estimates the independent components for each subject without taking others into account. The results of the “single session” were used to identify the components that corresponded to each subject’s noise, and these were eliminated (between four and five per subject), thus arriving at a more reliable database [30]. For the second level of analysis in MELODIC, “Multi-session Tensor-ICA” was used, which takes the entry data as a three-dimensional matrix (time, space, and subjects) and breaks it down into triplet, two-dimensional matrixes: time courses-ICA components and spatial maps-ICA components. The final observable product describes common components in all or most of the subjects and orders them according to the highest percentage of variation explained by the model, optimized by a distribution of non-Gaussian spatial sources, using fixed point iteration. The resulting components went through Pearson correlation tests and linear regressions were calculated; to determine the network dynamics and the modulation between networks [62], linear regression was used to measure the strength and direction of the linear relationship between two joint signals [63]. All these analyses were processed using our own written programs in MATLAB (Mathworks, Natick, MA, USA).

We used Inter-subject correlation analysis, which is highly comparable with GLM [64]. ISC analysis allows visualizing selective and time-locked activity across a wide network of brain areas, under some natural stimuli, comparing the whole neural response across all subjects, without a model [65]. We used the same data of the 23 subjects, normalized into an MNI coordinate system, spatially smoothed and realigned. The ISC analysis was performed using ISCtoolbox [66] (http://code.google.com/p/isc-toolbox/). Since ICA is a method that has been used mainly in blind signal separation unrelated to an external stimulus, in order to study the neural correlates of the extrinsic stimulus-evoked component of the brain activity, ISC was used to complement the analysis. For instance, inter-subject correlation has been proven effective in localizing cortical regions activated across subjects during free-viewing of movies [64], which in this study showed high consistency with our ICA results.

#### Cross correlation

The results of cross correlation were significant in most of comparisons p< 0.0125 (of a 0.05 Bonferroni corrected for 8 pairwise comparisons) with estimation error bounds 2.7 standard deviations from 0 (Fig 7), show a tendency for most of the networks to be one lag against the other. As it shows DMN vs C10, SMN vs DAN, DAN vs C12 and C7 vs C8, their highest value is in lag 0, however the next value is in lag -1 which indicates a tendency to be ahead, i.e. DMN tends to be ahead C10 but not in a fully way. Standing out among these findings is the SMN, which predict directly the group of networks on the left side (DMN), and in an inversely way those in the right column (DAN) (Fig 8). These two networks positively predict other networks, whose areas include posterior cingulate gyrus, lingual gyrus and precuneus; b) anterior cingulate cortex, lingual gyrus, postcentral gyrus and posterior insula; and c) SMN, whose connectivity changed immediately after the animal-slaughtering scenes were shown. On the other hand, areas of networks with increased activity during scenes that predated the time of death were a) medial frontal gyrus, precuneus, medial temporal gyrus and fusiform gyrus; and b) lingual gyrus, supramarginal gyrus and postcentral gyrus.

#### Behavioral data

To test whether the video caused aversive psychological effects, behavioral data was analyzed with the Wilcoxon signed-rank test comparing stress measures between t_1_ and t_2_.

### Neutral movie condition

#### Participants

The participants were 31 students (21 women, 10 men; *M* age = 20.90 yr., *SD* = 5.32) from the Psychology Department of Universidad de Guanajuato, who gave their informed consent to being part of the study. One participant lacking complete data was omitted (final n = 32).

#### Psychological measures

Perceived stress was measured with the pencil-paper version of the Stress and Activation Adjectives Checklist.

#### Neutral stimuli

The subjects watched 17 neutral fragments of the same movie projected in the previous study with 4 min and 53 sec length (1–17 x 16 s). This neutral version of the movie was re-edited and implemented thorough Java software. The neutral movie clips contained colored emotionally neutral scenes (e.g. hands, one house, sheep and cows in their ordinary context and scenes of people in abstract or trivial circumstances).

#### Procedure

Perceived stress was tested before (t_1_) and after viewing (t_2_) the neutral fragments. All video stimuli were presented via a video projection system in a semi-darkened experimental room isolated from noise and distractions. This system projected a 1.2 x 1.8 image onto a reflexive white screen. For all experimental stimuli, the picture took up 80% of the screen on a black background. The stimuli were evaluated in one single experimental session and 15 minutes of length.

#### Data analysis

Behavioral data for the neutral movie condition was analyzed with the Wilcoxon signed-rank test comparing stress measures between t_1_ and t_2_, same as in the fMRI movie condition.
